# Antihyperglycemic and antihyperlipidemic activities of aqueous extract of *Hericium erinaceus* in experimental diabetic rats

**DOI:** 10.1186/1472-6882-13-253

**Published:** 2013-10-03

**Authors:** Bin Liang, Zhengdong Guo, Fang Xie, Ainong Zhao

**Affiliations:** 1 Department of Clinical Laboratory, High Vocational Technological College, China Medical University, North Bei’er Road, No. 92, Shenyang 110001, Liaoning, PR China

**Keywords:** Animal model, Diabetes, Blood glucose, Oxidative stress

## Abstract

**Background:**

*Hericium erinaceus,* as a commonly used medicine or food, has attracted much attention due to its health effects when used as a home remedy for some diseases. The aim of this work was to investigate the hypoglycemic and hypolipidemic effects of aqueous extract of *Hericium erinaceus* (AEHE) in streptozotocin (STZ)-induced diabetic rats.

**Methods:**

Diabetes was induced in Wistar rats by the administration of STZ (55 mg/kg BW.) intraperitoneally. AEHE (100 and 200 mg/kg BW.) was administered for a period of 28 days. The effects of AEHE on glucose, insulin, and lipid files in blood, and oxidative stress parameters in the liver were evaluated. The body weights of rats were recorded at day 0, 14 and 28th days.

**Results:**

The administration of AEHE for 28 days in STZ diabetic rats resulted in a significant decrease in serum glucose level and a significant rise in serum insulin level. AEHE treatment attenuated lipid disorders. In addition, AEHE administration increased the activities of CAT, SOD, and GSH-Px, and GSH level, and reduced MDA level in the liver tissue significantly.

**Conclusion:**

Our results suggest that AEHE possesses hypoglycemic, hypolipidemic, and antioxidant properties in STZ-induced diabetes rats.

## Background

The worldwide prevalence of diabetes for all age groups was estimated to be 2.8% in 2000 and it is projected to be 4.4% in 2030 [1]. Especially in developed and developing countries, type 2 diabetes mellitus is now considered a worldwide epidemic, and is characterized by defects in both insulin secretion and insulin action that causes a chronic hyperglycaemic state [2]. A long-term metabolic disorder of carbohydrate metabolism is one of the most important causes of complications, such as angiopathy, neuropathy, retinopathy, deficiency in the antioxidant defense system, and lipid profile disorders [3-5].

There is considerable evidence demonstrating that oxidative stress caused by the production of free radical is a recognized participant in the development and progression of diabetes and its complications [6]. Hyperglycemia induces non-enzymatic glycosylation and activation of the polyol pathway, resulting in overproduction of reactive oxygen species (ROS) that lead to structural damages of liver, kidney, and pancreas [7]. These free radicals also destroy pancreatic β-cells that produce and secrete insulin [8]. There are many protective enzymes against ROS, such as superoxide dismutase (SOD), glutathione peroxidase (GPx) and catalase (CAT). Therefore, antioxidants have been considered as the treatment in diabetes.

*Hericium erinaceus,* as a commonly used medicine or food, has attracted much investigation due to its health effects when used as a home remedy for some diseases. Its fruiting bodies and the fungal mycelia exhibit various pharmacological activities, including anti-tumor [9], hemagglutinating [10], anti-microbial [11], immunomodulatory [12], anti-aging [13], and antioxidant activities [14].

Recently, *Hericium erinaceus* has been reported to significantly decrease lipid peroxidation level and increase antioxidant enzymes activities in experimental animals [15]. Yang BK, *et al.* reported that an exo-biopolymer produced from a submerged mycelial culture of *Hericium erinaceus* possesses hypolipidemic effect in dietary-induced hyperlipidemic rats [16], but the effects of *Hericium erinaceus* on hyperglycaemia, hyperlipidemia, lipid peroxidation and antioxidant enzymes activities in diabetes have not yet been examined. The present investigation was aimed to study the possible antihyperglycemic, antihyperlipidemic, and antioxidant effect of aqueous extract from *Hericium erinaceus* (AEHE) in STZ-induced diabetic rats.

## Methods

### Preparation of aqueous extract of *Hericium erinaceus*

*Hericium erinaceus* powder was purchased from Shanghai Kangzhou Company, People’s Republic of China. Streptozotocin and glibenclamide were purchased from Sigma Chemical Company (Shenyang, China). All chemicals and solvents used were of high purity and analytical grade.

#### Animals

The normoglycemic male Wistar rats (160-180 g) were used as the animal model. The rats were obtained from the Animal Center of China Medical University. The rats were kept in polypropylene cages under controlled temperature, humidity, and 12 h/12 h light/dark cycles, and were fed standard LAD 1000 M Rodent purified diet (GB 14924.3-2010, Trophic Anima Feed High-tech Co Ltd, China) and water *ad libitum*. All experiments complied with the Guidelines on Ethical Standards for the investigation in animals; the study was approved by China Medical University Committee for the care and use of laboratory animals.

#### Induction of experimental diabetes

Diabetes was induced by a single intraperitoneal injection of freshly prepared STZ [55 mg/kg body weight (b.w.)] in 0.1 M citrate buffer (pH 4.5), and subsequently allowed to drink 5% glucose solution overnight to overcome the drug-induced hypoglycemic mortality. Rats with a fasting plasma glucose ≥250 mg/dl on the third day after the STZ injection were considered diabetic and used for the study.

#### Animal experiment design

A total of 48 rats (32 diabetic rats, 16 normal rats) were used in the experiment. The rats were divided into six groups of eight each. An oral administration of the AEHE (100 and 200 mg/kg b.w.) and glibenclamide (30 mg/kg b.w.) were given for 28 consecutive days. The body weights of rats were recorded at day 0, 14 and 28th days.

The experimental groups (8 rats / group) were as follows:

Group I (NM group), normal control rats treated with vehicle alone.

Group II (NM + AEHE group), normal control rats treated with AEHE (100 mg/kg b.w.).

Group III (DM group), diabetic control rats treated with vehicle alone.

Group IV (DM + LAEHE group), diabetic rats were given AEHE (100 mg/kg b.w.).

Group V (DM + HAEHE group), diabetic rats were given AEHE (200 mg/kg b.w.).

Group VI (DM + Gli group), diabetic rats were given glibenclamide (30 mg/kg b.w.).

At the end of the experiment, the rats were anesthetized and sacrificed by decapitation. The liver were sampled and stored at −80°C till use.

#### Measurement of blood glucose and insulin

On day 0 (just after last treatment of STZ), 14, and 28th, blood samples in all groups were collected, and immediately centrifuged at 3000 × g for 20 min to obtain serum for biochemical estimations. The blood glucose was evaluated by glucose oxidase - peroxidase method (Zhongsheng Clinical Reagent Co., Ltd, Beijing, China). Serum insulin levels were measured by ELISA method using a commercial kit (Millipore, China).

#### Measurement of serum lipid files

At the end of the experiment, serum levels of total cholesterol (TC), high density lipoprotein cholesterol (HDL-C), low density lipoprotein cholesterol (LDL-C), and triglyceride (TG) were determined using commercially available kits (Zhongsheng Clinical Reagent Co., Ltd, Beijing, China).

#### Measurement of oxidative stress parameters in liver

Liver of rats were removed and placed in 10% KCl (10 ml/g tissue), and homogenized on ice for 120 s with a DY89-II homogenizer (Ningbo Scientz Biotechnology Co. Ltd. China) at 600 rpm. Tissue homogenates were centrifuged at 1000 × g at 4°C for 10 min to remove tissue debris, and clear supernatant was used for further analyses. The superoxide dismutase (SOD), glutathione peroxidase (GSH-Px) and catalase (CAT) activities, glutathione (GSH) and malondialdehyde (MDA) in liver samples were measured using commercially available kits (Jiancheng Bioengineering Institute, Nanjing, China).

#### Statistical analysis

Statistical analyses were performed using SPSS15.0 (SPSS, Chicago, IL). All the experimental data were expressed as mean ± standard deviation (SD). For significance verification by groups, one-way or two-way ANOVA was performed, followed by Tukey’s test. Two-way repeated measures ANOVA was used to examine the overall effects of treatment and time on the change in blood glucose and insulin levels. A *P* value of <0.05 was considered significant.

## Results

### Effect of AEHE on body weights of rats

The body weight was recorded at day 0, 14 and 28th and the final data were shown in Table 1. The body weights of diabetic rats were similar to that of normal controls just after STZ injection (*P* > 0.05). The bodyweights of the diabetic control rats were significantly lower compared to normal control group at day 14 and 28th (*P* < 0.05). The diabetic control rats gained less body weights than normal control group throughout the experiment. But, body weight gains were significantly increased in rats administrated with AEHE and glibenclamide, respectively.

**Table 1:** Effect of AEHE on body weights in experimental rats

**Days**	**NM**	**NM + AEHE**	**DM**	**DM + LAEHE**	**DM + HAEHE**	**DM + Gli**
Day 0 Body weights (g)	169.3 ± 6.2	170.6 ± 6.9	172.4 ± 6.0	174.1 ± 6.5	169.6 ± 7.1	173.0 ± 6.8
Day 14 Body weights (g)	211.6 ± 12.1	207.0 ± 10.0	181.1 ± 9.4^a^	193.5 ± 13.1^a,b^	203.3 ± 12.6^a,b^	209.4 ± 12.9^b^
Day 28 Body weights (g)	302.3 ± 8.6	297.8 ± 11.0	226.3 ± 9.8^a^	251.0 ± 10.2^a,b^	291.3 ± 12.1^b^	292.2 ± 10.5^b^

Values are expressed as mean ± SD in each group. *NM group*: normal control rats treated with vehicle alone; *NM + AEHE group*: normal control rats treated with AEHE (100 mg/kg b.w.); *DM group*: diabetic control rats treated with vehicle alone; *DM + LAEHE group*: diabetic rats were given AEHE (100 mg/kg b.w.); *DM + HAEHE group*: diabetic rats were given AEHE (200 mg/kg b.w.); *DM + Gli group*: diabetic rats were given glibenclamide (30 mg/kg b.w.). ^a^indicates statistical significance of *P* < 0.01 compared to the NM group; ^b^indicates statistical significance of *P* < 0.01 compared to the DM group.

### Effect of AEHE on levels of blood glucose and insulin

Table 2 depicts the serum glucose levels in normal and diabetic rats supplemented with AEHE and glibenclamide. STZ-treated diabetic rats showed significant increase in the levels of blood glucose when compared to normal rats (*P* < 0.05). Serum glucose level was measured in normal and diabetes rats on day 0, 14, and 28th of drug treatment. After treatment with AEHE at 100 and 200 mg/kg b.w. the blood glucose levels on day 14 and 28th were significantly reduced compared to those on day 0 (*P* < 0.01). The glibenclamide treated rats also showed significant reduction in serum glucose level (*P* < 0.05). Therefore, AEHE and glibenclamide administration attenuated hyperglycemia observed in the rats, while no significant changes were observed in NM, NM + AEHE, and DM groups (*P* > 0.05). Moreover, at the same day, serum glucose in AEHE and glibenclamide treated rats were significantly reduced when compared with diabetes control rats (*P* < 0.05).

**Table 2:** Effect of AEHE on serum glucose levels in rats during the experiment days

	**NM**	**NM + AEHE**	**DM**	**DM + LAEHE**	**DM + HAEHE**	**DM + Gli**
Day 0	105.6 ± 7.8	109.3 ± 8.6	278.9 ± 11.2^a^	286.3 ± 9.4^a^	291.0 ± 11.9^a^	289.1 ± 10.3^a^
Day 14	106.1 ± 8.1	103.4 ± 7.2	289.7 ± 10.4^a^	226.5 ± 8.7^a,b,c^	199.6 ± 12.5^a,b,c^	202.3 ± 8.5^a,b,c^
Day 28	107.3 ± 7.0	98.3 ± 9.9	296.1 ± 8.8^a^	163.2 ± 9.6^a,b,c^	135.4 ± 10.9^a,b,c^	129.3 ± 8.3^a,b,c^

Values are expressed as mean ± SD in each group. *NM group*: normal control rats treated with vehicle alone; *NM + AEHE group*: normal control rats treated with AEHE (100 mg/kg b.w.); *DM group*: diabetic control rats treated with vehicle alone; *DM + LAEHE group*: diabetic rats were given AEHE (100 mg/kg b.w.); *DM + HAEHE group*: diabetic rats were given AEHE (200 mg/kg b.w.); *DM + Gli group*: diabetic rats were given glibenclamide (30 mg/kg b.w.). ^a^indicates statistical significance of *P* < 0.05 compared to the NM group; ^b^indicates statistical significance of *P* < 0.05 compared to the DM group. ^c^indicates statistical significance of *P* < 0.05 compared to the same group at day 0.

Data concerning the fasting serum insulin levels were presented in Table 3. After 28 day period, insulin level decreased significantly in DM group. But, both 100 mg/kg b.w. and 200 mg/kg b.w. AEHE groups showed higher levels of insulin at day 14 and day 28 significantly compared with the diabetic control (*P* < 0.05), and the glibenclamide treated rats also showed significant increase in insulin level (*P* < 0.05). In addition, 100 mg/kg b.w. AEHE itself did not influence insulin level of rats in NM + AEHE group. Furthermore, no interaction effects (treatment × time) were seen on insulin levels (*P* > 0.05).

**Table 3:** Effect of AEHE on insulin levels in rats during the experiment days

	**NM**	**NM + AEHE**	**DM**	**DM + LAEHE**	**DM + HAEHE**	**DM + Gli**
Day 0	20.4 ± 1.0	21.1 ± 1.3	9.6 ± 0.9^a^	9.8 ± 0.9^a^	10.1 ± 1.1^a^	9.9 ± 0.9^a^
Day 14	21.2 ± 1.2	21.6 ± 1.2	9.4 ± 1.1^a^	12.4 ± 1.2^a,b,c^	16.3 ± 1.3^a,b,c^	15.8 ± 1.2^a,b,c^
Day 28	20.9 ± 1.2	22.4 ± 1.1	9.7 ± 1.2^a^	16.4 ± 1.1^a,b,c^	18.9 ± 1.4^a,b,c^	18.6 ± 1.6^a,b,c^

Values are expressed as mean ± SD in each group. *NM group*: normal control rats treated with vehicle alone; *NM + AEHE group*: normal control rats treated with AEHE (100 mg/kg b.w.); *DM group*: diabetic control rats treated with vehicle alone; *DM + LAEHE group*: diabetic rats were given AEHE (100 mg/kg b.w.); *DM + HAEHE group*: diabetic rats were given AEHE (200 mg/kg b.w.); *DM + Gli group*: diabetic rats were given glibenclamide (30 mg/kg b.w.). ^a^indicates statistical significance of *P* < 0.05 compared to the NM group; ^b^indicates statistical significance of *P* < 0.05 compared to the DM group, ^c^indicates statistical significance of *P* < 0.05 compared to the same group at day 0.

### Effect of AEHE on levels of serum lipid files

At the end of the experiment the levels of serum TC, TG, HDL-C and LDL-C in different experimental groups were shown in Table 4. The results showed that serum TG, TC and LDL-C levels were significantly increased (*P* <0.05), whereas the serum HDL-C level was significantly decreased in DM group as compared to NM group (*P* < 0.05). After the administration of AEHE (200 mg/kg b.w.) for 28 days, a significant decrease in TG, TC, and LDL-C, and a significant increase in HDL-C were observed in DM + HAEHE group compared with DM group (*P* < 0.05). Moreover, after the administration of AEHE (100 mg/kg b.w.) for 28 days, the alteration in lipid metabolism was partially attenuated as evidenced by decreased serum TG and TC levels in DM + LAEHE group when compared with DM group (*P* < 0 .05).

**Table 4:** Effect of AEHE on levels of serum TC, TG, HDL-C and LDL-C in experimental rats

	**NM**	**NM + AEHE**	**DM**	**DM + LAEHE**	**DM + HAEHE**	**DM + Gli**
TG (mmol/L)	1.39 ± 0.31	1.41 ± 0.39	2.51 ± 0.36^a^	2.01 ± 0.41^b^	1.46 ± 0.37^c^	1.42 ± 0.31^c^
TC (mmol/L)	1.67 ± 0.29	1.72 ± 0.35	2.69 ± 0.32^a^	2.13 ± 0.41^c^	1.71 ± 0.31^c^	1.72 ± 0.34^c^
HDL-C (mmol/L)	1.24 ± 0.25	1.29 ± 0.29	0.89 ± 0.29^a^	1.01 ± 0.20	1.19 ± 0.23^b^	1.22 ± 0.26^b^
LDL-C (mmol/L)	0.54 ± 0.07	0.61 ± 0.09	0.81 ± 0.12^a^	0.77 ± 0.09	0.65 ± 0.06^c^	0.59 ± 0.08^c^

Values are expressed as mean ± SD in each group. *NM group*: normal control rats treated with vehicle alone; *NM + AEHE group*: normal control rats treated with AEHE (100 mg/kg b.w.); *DM group*: diabetic control rats treated with vehicle alone; *DM + LAEHE group*: diabetic rats were given AEHE (100 mg/kg b.w.); *DM + HAEHE group*: diabetic rats were given AEHE (200 mg/kg b.w.); *DM + Gli group*: diabetic rats were given glibenclamide (30 mg/kg b.w.). ^a^indicates statistical significance of *P* < 0.05 compared to the NM group; ^b^indicates statistical significance of *P* < 0.05 compared to the DM group. ^c^indicates statistical significance of *P* < 0.01 compared to the DM group.

### Effect of AEHE on levels of oxidative stress parameters in liver tissue

**Figure 1:**
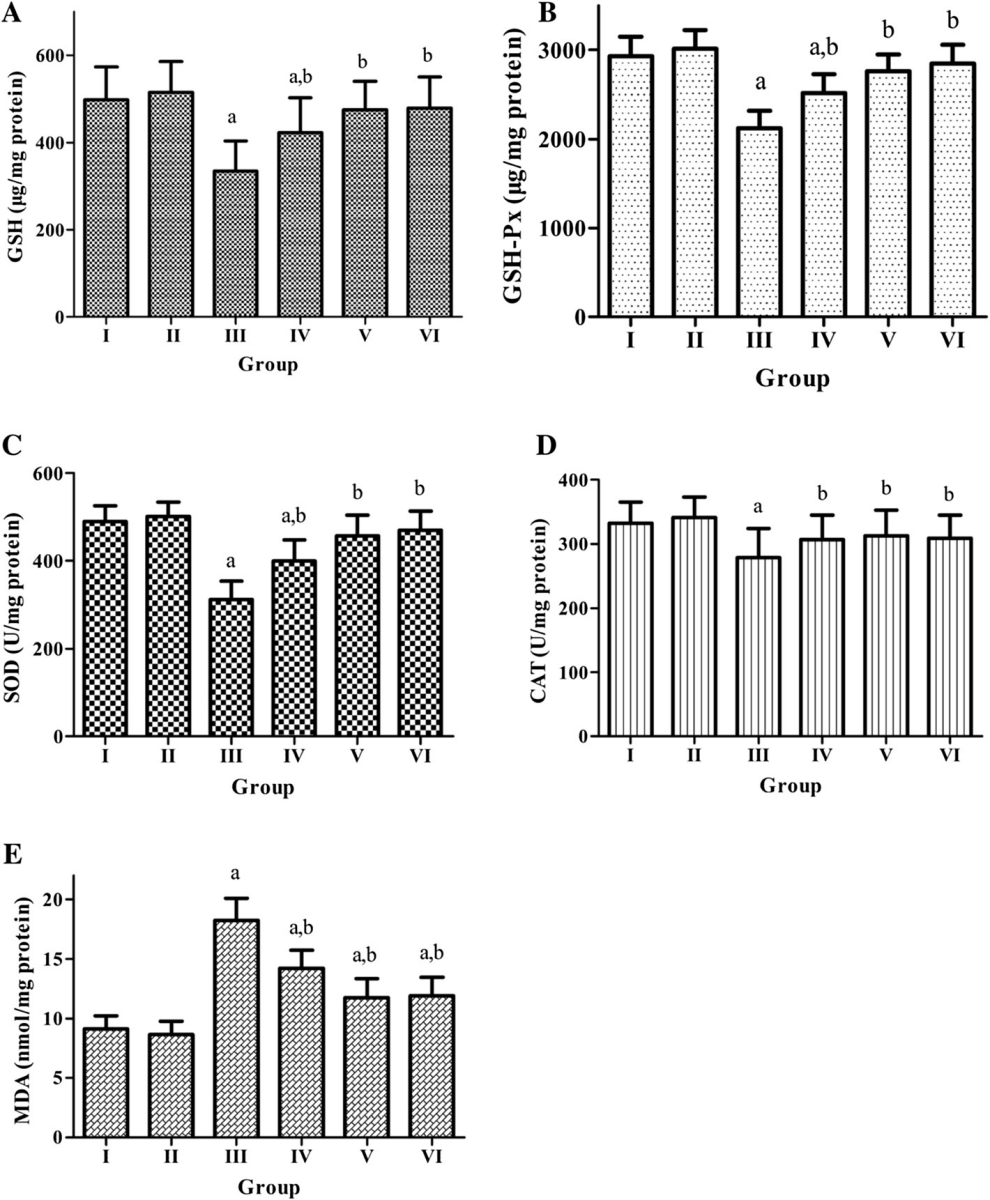
**Effect of AEHE treatment on hepatic oxidative stress markers in experimental rats. (A)** glutathione levels (GSH levels, μg/mg protein), **(B)** glutathione peroxidase levels (GSH-Px levels, μg/mg protein ), **(C)** superoxide dismutase activity (SOD activity, U/mg protein), **(D)** catalase activity (CAT activity, U/mg protein), and **(E)** malondialdehyde levels (MDA levels, μg/mg protein). Group I: normal control rats treated with vehicle alone; Group II: normal control rats treated with AEHE (100 mg/kg b.w.); Group III: diabetic control rats treated with vehicle alone; Group IV: diabetic rats were given AEHE (100 mg/kg b.w.); Group V: diabetic rats were given AEHE (200 mg/kg b.w.); Group VI: diabetic rats were given glibenclamide (30 mg/kg b.w.). a indicates statistically different from NM group, b indicates statistically different from DM group.

Figure 1(A-E) showed the activities of enzymatic antioxidants SOD, CAT, GSH-Px, protein antioxidant GSH, and lipid peroxidation product MDA in the liver of experimental rats. A significant decrease of the activities of SOD, CAT, GSH-Px, the level of GSH, and an increase of MDA in the liver tissue were observed in the DM group compared with NM group (*P* <0 .05). The level of GSH and the activities of SOD, CAT, GSH-Px were significantly restored in the AEHE treated diabetic rats (DM + LAEHE group and DM + HAEHE group) (*P* < 0.05). However, AEHE supplementation did not alter the level of GSH and the activities of SOD, CAT, GSH-Px significantly in NM + AEHE group (*P* > 0.05). In addition, AEHE administration significantly lowered MDA level in the diabetic rats (*P* <0 .05), while no effect was observed in the normal control rats (*P* > 0.05).

## Discussion

In diabetes mellitus, chronic hyperglycemia produces multiple biochemical abnormalities [17,18], and studies in both humans and animal models clearly implicate the contribution of oxidative stress to the pathogenesis of diabetes [19-21]. Clinical studies have demonstrated that tight control of hyperglycaemia can reduce the occurrence or progression of diabetes; however, with the current hypoglycemic or antidiabetic drugs, it is difficult to achieve and/or maintain tight glycemic control in diabetic patients [22-24]. Many studies indicate that multiple drugs are required to achieve optimal glycemic target in many diabetic patients. Currently, one of such complementary options is the potential of “concurrently targeting hyperglycemia and oxidative stress” [25]. Therefore, the use of alternate therapies that specifically target oxidative stress implicated in diabetes may be advantageous in addition to a strict glucose control.

In China, many herbs have been used to treat diabetes. A total of more than 400 species were reported to display hypoglycemic effects [26], and *Hericium erinaceus* and its components have attracted interest in the medical research during the past two decades because of its various biological and clinical properties, as well as its antioxidant activity. *Hericium erinaceus* contains polysaccharides, oligosaccharide, sterol, fatty acid, erinacine, hericenone, and so on. Wang HX, *et al.* found that a laccase with inhibitory activity toward HIV-1 reverse transcriptase, was isolated from *Hericium erinaceus*[27]*.* Nagai K, *et al.* reported that dilinoleoyl-phosphatidylethanolamine extracted from *Hericium erinaceus* was one of the molecules effective at reducing ER-stress dependent cell death in the mouse neuroblastoma cell line [28]. Kim SP, *et al.* demonstrated that the extracts from *Hericium erinaceus* against bacterium infection in mice occur through the activation of innate immune cells [12]. Han ZH, *et al.* reported that *Hericium erinaceus* can significantly decrease lipid peroxidation level and increase antioxidant enzymes activities in experimental animals [15]. Many studies have been performed for examining its pharmacological function in many diseases. In this study, we investigated the antidiabetic and antioxidant effects of AEHE in STZ-induced diabetic model rats compared with glibenclamide treated rats, which was diabetic control group, and evaluated the possible function of AEHE as a complementary medicine on glycemic control. Glibenclamide, a standard hypoglycemic drug, has been widely used for many years to treat diabetes by promoting insulin secretion through blockade of ATP-dependent potassium channels in the pancreatic β-cells [29]. Our results in the study indicate that the still insulin producing cells appear to be functional in releasing insulin responsible for most of the metabolic effects. The effect of AEHE in our study is similar to that of glibenclamide, which suggested the mechamism of AEHE may be also similar to that of glibenclamide.

STZ-induced diabetic rats are one of the animal models of human insulin-dependent diabetes mellitus [30]; these rats are characterized by high fasting blood glucose levels and drastic reduction in blood insulin concentration [31]. In our study, serum glucose levels were measured on day 0, 14 and 28th. Till day 28th, serum glucose levels were significantly decreased compared with those obtained on day 0 and 14th. All results showed that the daily administration of the AEHE during 28 days abolished the blood glucose increase in the STZ- induced diabetic rats, and this effect was dose dependent. Moreover, administration of AEHE to diabetic rats resulted in a significant increase in insulin levels. The possible mechanism by which AEHE exhibits antihyperglycemic action in diabetic rats may be due to the pancreatic production of insulin from the existing β-cells or by its release from the bound form.

Several studies also have demonstrated that the hypoglycemic or antidiabetic effect of some natural herb extracts can be attributed to their insulin-trophic effect that enables the reduction of blood glucose levels, liver glycogen content, and serum lipids through the control of serum insulin [32,33]. Similarly, our results also show that AEHE administration increased insulin production.

Diabetic rats showed marked reduction in their body weights compared to normal rats, which could be due to the degradation of structural proteins. The excessive catabolism of protein to provide amino acids for gluconeogenesis during insulin deficiency resulted in muscle wasting and weight loss in diabetic untreated rats. STZ, as a highly cytotoxic agent of pancreatic β-cells, induces diabetes by damaging the cells that causes the reduction in insulin release [34]. Our data indicate that rise in insulin levels upon treatment with AEHE in diabetic rats resulted in improved glycemic control, which prevented the loss of body weight.

Abnormality in lipid metabolism in diabetes, which are often important determinants of the course and status of the diseases, are characterized by increase in TC, TG, LDL-C and fall in HDL-C [34]. Moreover, Ghoul JE, *et al.* have demonstrated that the deficiency in insulin or the insulin resistance may be responsible for hyperlipidaemia due to the insulin inhibiting action on the key enzyme in the cholesterol biosynthesis [35]. In our study, we recorded a significant increase in the serum levels of TG, TC and LDL-C, and a significant decrease in the serum level of HDL-C in STZ-induced diabetic rats; AEHE administration decreased serum TG, TC and LDL-C levels, and increased HDL-C level in STZ-induced diabetic rats. It indicates that AEHE treatment is useful to normalize the lipid profile by regulating blood glucose and insulin.

Oxidative stress in diabetes coexists with a decrease in the antioxidant status [36], and hyperglycemia is known to accentuate oxidative stress in liver [37]. The elevated generation of ROS and the simultaneous decline in antioxidative defence mechanisms observed in diabetic patients could promote the development of neuropathy, nephropathy, retinopathy and vascular disorders [38]. Our results demonstrated that the activities of antioxidant enzymes (SOD, CAT and GSH-Px), and antioxidant (GSH) in the liver of the diabetic rats were significantly lower than those in the normal control group. However, they were restored in AEHE treated diabetic rats, implying that AEHE could scavenge oxygen free radicals. MDA is a reliable marker of lipid peroxidation. Our results showed that MDA level was significantly increased in the liver of untreated diabetic rats, and AEHE treatment attenuated lipid peroxidation. Moreover, increased MDA level in the liver clearly indicates the role of oxidative stress in diabetes, and AEHE might reduce lipid peroxidation by modulating glucose/insulin system. To sum up, AEHE has direct or indirect antidiabetogenic effects by decreasing oxidative stress, and this may be one of the reasons that AEHE improves glycometabolism. Thus, the present investigation showed that AEHE possess potent antioxidant activity, which may be responsible for its hypoglycemic and hypolipidemic properties.

## Conclusion

According to our present findings, AEHE possessed a significant anti-hyperglycemic and anti-hyperlipidemic effect in STZ- induced diabetic rats. AEHE also can restore the antioxidant status in treated diabetic rats. However, further research is needed to gain a better understanding of its potential therapeutic action, the implicated phytochemical constituents and the exact mechanism of action.

## Abbreviations

AEHE: Aqueous extract of *hericium erinaceus*; ROS: Reactive oxygen species; SOD: Superoxide dismutase; GPx: Glutathione peroxidase; CAT: Catalase; MDA: Malondialdehyde.

## Competing interests

The authors declare that they have no competing interests.

## Authors’ contributions

LB and GZ made substantial contributions to conception and design, and helped to draft the manuscript. LB and XF collected and interpreted the experimental data. ZA performed the statistical analysis. All authors read and approved the final manuscript.

## Pre-publication history

The pre-publication history for this paper can be accessed here:

http://www.biomedcentral.com/1472-6882/13/253/prepub

## References

[B1] Wild S Roglic G Green A Sicree R King H Global prevalence of diabetes: estimates for the year 2000 and projections for 2030 Diabetes Care 2004 27 5 1047 1053 10.2337/diacare.27.5.1047 15111519

[B2] Zimmet P Alberti KG Shaw J Global and societal implications of the diabetes epidemic Nature 2001 414 6865 782 787 10.1038/414782a 11742409

[B3] Cheung N Mitchell P Wong TY Diabetic retinopathy Lancet 2010 376 9735 124 136 10.1016/S0140-6736(09)62124-3 20580421

[B4] Goldfine AB Fonseca V Management of diabetes mellitus in patients with cardiovascular disease in the Bypass Angioplasty Revascularization Investigation 2 Diabetes (BARI 2D) trial Circulation 2010 121 22 2447 2449 10.1161/CIRCULATIONAHA.109.925883 20530022

[B5] Kotseva K Wood D De Backer G De Bacquer D Pyorala K Reiner Z Keil U EUROASPIRE III. Management of cardiovascular risk factors in asymptomatic high-risk patients in general practice: cross-sectional survey in 12 European countries Eur J Cardiovasc Prev Rehabil 2010 17 5 530 540 10.1097/HJR.0b013e3283383f30 20577089

[B6] Baynes JW Thorpe SR Role of oxidative stress in diabetic complications: a new perspective on an old paradigm Diabetes 1999 48 1 1 9 10.2337/diabetes.48.1.1 9892215

[B7] Ozkaya D Naziroglu M Armagan A Demirel A Koroglu BK Colakoglu N Kukner A Sonmez TT Dietary vitamin C and E modulates oxidative stress induced-kidney and lens injury in diabetic aged male rats through modulating glucose homeostasis and antioxidant systems Cell Biochem Funct 2011 29 4 287 293 10.1002/cbf.1749 21416480

[B8] Laybutt DR Kaneto H Hasenkamp W Grey S Jonas JC Sgroi DC Groff A Ferran C Bonner-Weir S Sharma A Increased expression of antioxidant and antiapoptotic genes in islets that may contribute to beta-cell survival during chronic hyperglycemia Diabetes 2002 51 2 413 423 10.2337/diabetes.51.2.413 11812749

[B9] Mizuno T Wasa T Ito H Suzuki C Ukai N Antitumor-active polysaccharides isolated from the fruiting body of Hericium erinaceum, an edible and medicinal mushroom called yamabushitake or houtou Biosci Biotechnol Biochem 1992 56 2 347 348 10.1271/bbb.56.347 1368310

[B10] Gong M An J Lu HZ Wu CF Li YJ Cheng JQ Bao JK Effects of denaturation and amino acid modification on fluorescence spectrum and hemagglutinating activity of Hericium erinaceum Lectin Acta Biochim Biophys Sin 2004 36 5 343 350 10.1093/abbs/36.5.343 15156276

[B11] Yim MH Shin JW Son JY Oh SM Han SH Cho JH Cho CK Yoo HS Lee YW Son CG Soluble components of Hericium erinaceum induce NK cell activation via production of interleukin-12 in mice splenocytes Acta Pharmacol Sin 2007 28 6 901 907 10.1111/j.1745-7254.2007.00577.x 17506950

[B12] Kim SP Moon E Nam SH Friedman M Hericium erinaceus mushroom extracts protect infected mice against Salmonella Typhimurium-Induced liver damage and mortality by stimulation of innate immune cells J Agric Food Chem 2012 60 22 5590 5596 10.1021/jf300897w 22624604

[B13] Zhang Z Lv G Pan H Pandey A He W Fan L Antioxidant and hepatoprotective potential of endo-polysaccharides from Hericium erinaceus grown on tofu whey Int J Biol Macromol 2012 51 5 1140 1146 10.1016/j.ijbiomac.2012.09.002 22982810

[B14] Malinowska E Krzyczkowski W Lapienis G Herold F Improved simultaneous production of mycelial biomass and polysaccharides by submerged culture of Hericium erinaceum: optimization using a central composite rotatable design (CCRD) J Ind Microbiol Biotechnol 2009 36 12 1513 1527 10.1007/s10295-009-0640-x 19784853

[B15] Han ZH Ye JM Wang GF Evaluation of in vivo antioxidant activity of Hericium erinaceus polysaccharides Int J Biol Macromol 2013 52 66 71 23000690 10.1016/j.ijbiomac.2012.09.009

[B16] Yang BK Park JB Song CH Hypolipidemic effect of an Exo-biopolymer produced from a submerged mycelial culture of Hericium erinaceus Biosci Biotechnol Biochem 2003 67 6 1292 1298 10.1271/bbb.67.1292 12843656

[B17] Giugliano D Ceriello A Paolisso G Oxidative stress and diabetic vascular complications Diabetes Care 1996 19 3 257 267 10.2337/diacare.19.3.257 8742574

[B18] Rajasekaran S Sivagnanam K Subramanian S Antioxidant effect of Aloe vera gel extract in streptozotocin-induced diabetes in rats Pharmacol Rep 2005 57 1 90 96 15849382

[B19] Nishikawa T Edelstein D Du XL Yamagishi S Matsumura T Kaneda Y Yorek MA Beebe D Oates PJ Hammes HP Normalizing mitochondrial superoxide production blocks three pathways of hyperglycaemic damage Nature 2000 404 6779 787 790 10.1038/35008121 10783895

[B20] Giacco F Brownlee M Oxidative stress and diabetic complications Circ Res 2010 107 9 1058 1070 10.1161/CIRCRESAHA.110.223545 21030723 PMC2996922

[B21] Forbes JM Coughlan MT Cooper ME Oxidative stress as a major culprit in kidney disease in diabetes Diabetes 2008 57 6 1446 1454 10.2337/db08-0057 18511445

[B22] Dronavalli S Duka I Bakris GL The pathogenesis of diabetic nephropathy Nat Clin Pract Endocrinol Metab 2008 4 8 444 452 10.1038/ncpendmet0894 18607402

[B23] The Diabetes Control and Complications Trial Research Group The effect of intensive treatment of diabetes on the development and progression of long-term complications in insulin-dependent diabetes mellitus N Engl J Med 1993 329 14 977 986 8366922 10.1056/NEJM199309303291401

[B24] Ohkubo Y Kishikawa H Araki E Miyata T Isami S Motoyoshi S Kojima Y Furuyoshi N Shichiri M Intensive insulin therapy prevents the progression of diabetic microvascular complications in Japanese patients with non-insulin-dependent diabetes mellitus: a randomized prospective 6-year study Diabetes Res Clin Pract 1995 28 2 103 117 10.1016/0168-8227(95)01064-K 7587918

[B25] Erejuwa OO Management of diabetes mellitus: could simultaneous targeting of hyperglycemia and oxidative stress be a better panacea? Int J Mol 2012 13 3 2965 2972 10.3390/ijms13032965 PMC3317697 22489136

[B26] Bailey CJ Day C Traditional plant medicines as treatments for diabetes Diabetes Care 1989 12 8 553 564 10.2337/diacare.12.8.553 2673695

[B27] Wang HX Ng TB A new laccase from dried fruiting bodies of the monkey head mushroom Hericium erinaceum Biochem Biophys Res Commun 2004 322 1 17 21 10.1016/j.bbrc.2004.07.075 15313167

[B28] Nagai K Chiba A Nishino T Kubota T Kawagishi H Dilinoleoyl-phosphatidylethanolamine from Hericium erinaceum protects against ER stress-dependent Neuro2a cell death via protein kinase C pathway J Nutr Biochem 2006 17 8 525 530 10.1016/j.jnutbio.2005.09.007 16426828

[B29] Sokolovska J Isajevs S Sugoka O Sharipova J Paramonova N Isajeva D Rostoka E Sjakste T Kalvinsh I Sjakste N Comparison of the effects of glibenclamide on metabolic parameters, GLUT1 expression, and liver injury in rats with severe and mild streptozotocin-induced diabetes mellitus Medicina (Kaunas) 2012 48 10 532 543 23324250

[B30] Bach JF Insulin-dependent diabetes mellitus as an autoimmune disease Endocr Rev 1994 15 4 516 542 7988484 10.1210/edrv-15-4-516

[B31] Burcelin R Eddouks M Maury J Kande J Assan R Girard J Excessive glucose production, rather than insulin resistance, accounts for hyperglycaemia in recent-onset streptozotocin-diabetic rats Diabetologia 1995 38 3 283 290 10.1007/BF00400632 7758874

[B32] Shen Y Fukushima M Ito Y Muraki E Hosono T Seki T Ariga T Verification of the antidiabetic effects of cinnamon (Cinnamomum zeylanicum) using insulin-uncontrolled type 1 diabetic rats and cultured adipocytes Biosci Biotechnol Biochem 2010 74 12 2418 2425 10.1271/bbb.100453 21150113

[B33] Juarez-Rojop IE Diaz-Zagoya JC Ble-Castillo JL Miranda-Osorio PH Castell-Rodriguez AE Tovilla-Zarate CA Rodriguez-Hernandez A Aguilar-Mariscal H Ramon-Frias T Bermudez-Ocana DY Hypoglycemic effect of Carica papaya leaves in streptozotocin-induced diabetic rats BMC Complement Altern Med 2012 12 1 236 10.1186/1472-6882-12-236 23190471 PMC3551835

[B34] Gupta S Sharma SB Bansal SK Prabhu KM Antihyperglycemic and hypolipidemic activity of aqueous extract of Cassia auriculata L. leaves in experimental diabetes J Ethnopharmacol 2009 123 3 499 503 10.1016/j.jep.2009.02.019 19473793

[B35] Ghoul JE Smiri M Ghrab S Boughattas NA Ben-Attia M Antihyperglycemic, antihyperlipidemic and antioxidant activities of traditional aqueous extract of Zygophyllum album in streptozotocin diabetic mice ISP 2012 19 1 35 42 10.1016/j.pathophys.2011.12.001 22209473

[B36] Picton SF Flatt PR McClenaghan NH Differential acute and long term actions of succinic acid monomethyl ester exposure on insulin-secreting BRIN-BD11 cells Int J Exp Diabetes Res 2001 2 1 19 27 10.1155/EDR.2001.19 12369722 PMC2478528

[B37] Opara EC Oxidative stress, micronutrients, diabetes mellitus and its complications J R Soc Promot Health 2002 122 1 28 34 10.1177/146642400212200112 11989140

[B38] Al-Azzawie HF Alhamdani MS Hypoglycemic and antioxidant effect of oleuropein in alloxan-diabetic rabbits Life Sci 2006 78 12 1371 1377 10.1016/j.lfs.2005.07.029 16236331

